# Supplementary material for: Metagenomic analysis of the cow, sheep, reindeer and red deer rumen
Source: Sci Rep. 2021 Jan 21;11:1990. doi: 10.1038/s41598-021-81668-9 (PMC7820578; doi:10.1038/s41598-021-81668-9)
Supplement: Supplementary file 11 — Supplementary Figure S1. [file 41598_2021_81668_MOESM11_ESM.pdf]

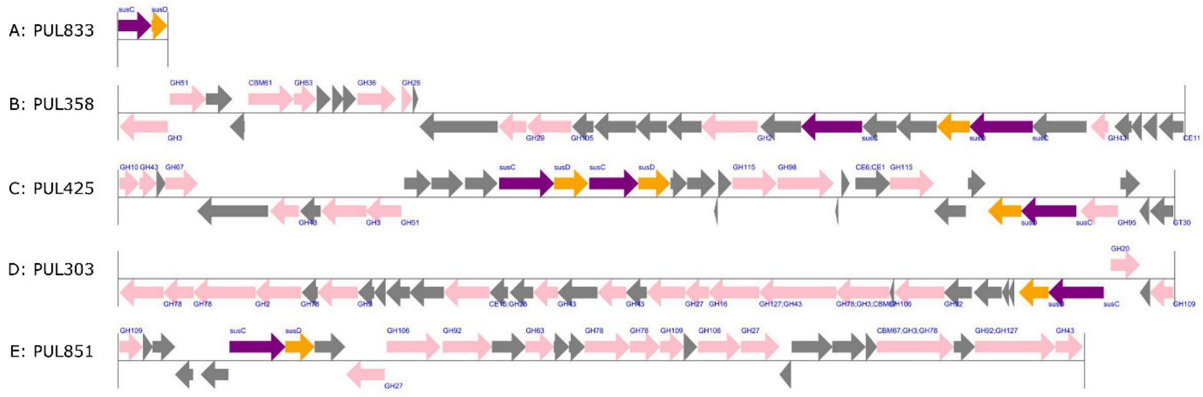

Supplementary fig 1: Several predicted polysaccharide utilisation loci (PULs). A: The most common PUL consisting of only a susC/D pair. B-E: The 4 largest PULs identified in our dataset.
